# Venous Thromboembolism Chemoprophylaxis in Knee Arthroscopy: A Break-Even Analysis of Cost

**DOI:** 10.1177/03635465221130990

**Published:** 2022-11-03

**Authors:** Brandon J. Martinazzi, Gregory J. Kirchner, F. Jeffrey Lorenz, Vincenzo Bonaddio, Shawn Hines, Raymond Y. Kim, Robert A. Gallo

**Affiliations:** † Penn State Health, Milton S. Hershey Medical Center, Department of Orthopaedics & Rehabilitation, Hershey, Pennsylvania, USA; Investigation performed at Penn State Health, Milton S. Hershey Medical Center, Hershey, Pennsylvania, USA

**Keywords:** economic and decision analysis, knee ligaments, venous thromboembolism

## Abstract

**Background:**

Symptomatic venous thromboembolism (VTE) is a serious and costly complication after knee arthroscopy. There continues to be debate regarding the use of VTE prophylaxis after knee arthroscopy, and minimal research has explored its cost-effectiveness.

**Hypothesis:**

Both aspirin and enoxaparin would be cost-effective in preventing symptomatic VTE.

**Study Design:**

Economic and decision analysis; Level of evidence, 3.

**Methods:**

The literature was searched and the TriNetX research database was queried to determine a range of initial rates of VTE. An open-access retail database was used to determine the mean retail price for aspirin (325 mg) and enoxaparin (30 mg and 40 mg). Our institutional records were used to determine the cost of treating VTE. A “break-even” analysis was then performed to determine the absolute risk reduction necessary to make these drugs cost-effective. This value was then used to calculate the number of patients who would need to be treated (NNT) to prevent a single VTE while still breaking even on cost.

**Results:**

The cost of treating VTE was $9407 (US Dollars). Aspirin (325 mg), enoxaparin (30 mg), and enoxaparin (40 mg) were found to cost $1.86, $188.72, and $99.99, respectively. The low, TriNetX, and high rates of VTE were 0.34%, 0.86%, and 10.9%, respectively. Aspirin was cost-effective at all 3 rates if the initial rate decreased by 0.02% (NNT = 5058). Both formulations of enoxaparin were cost-effective at the high initial rate if they decreased by 2.01% (NNT = 50) and 1.06% (NNT = 94), respectively. However, at the low and TriNetX rates, the 2 doses of enoxaparin were not cost-effective because their final break-even rate exceeded the initial VTE rate.

**Conclusion:**

Aspirin and, in some cases, enoxaparin are cost-effective treatments for VTE prophylaxis after knee arthroscopy.

Symptomatic venous thromboembolism (VTE) after knee arthroscopy compromises patient safety, increases unplanned health care encounters, and ultimately increases health care costs. VTE is diagnosed in 400,000 patients each year and carries a significant economic burden. Experts estimate that VTE costs the US health care system 7 to 10 billion dollars annually.^7^ Also, >60% of patients who develop a VTE require hospitalization.^5^ Among knee arthroscopy patients, the rate of symptomatic VTE has been reported^21^ as high as 10.9%. VTE prevention after knee arthroscopy is important in improving patient outcomes and reducing costs.

Most knee surgeons utilize sequential compression devices, early ambulation, and chemoprophylaxis as a multimodal approach to VTE prophylaxis.^21^ Low molecular weight heparin (LMWH) and aspirin are commonly used and have proven efficacy in preventing VTE.^2,15,18,21^ Alternative chemoprophylactic agents that are increasingly used include factor Xa inhibitors.^19^ Nevertheless, some studies have suggested that chemoprophylaxis to prevent VTE may not be necessary.^3,19^ However, forgoing VTE chemoprophylaxis remains controversial, and some authors argue that the use of chemoprophylaxis should be limited to high-risk patients.^12^

Cost-effectiveness has been called into question within the ongoing debate regarding the utility of VTE chemoprophylaxis after knee arthroscopy.^26^ Knowledge remains extremely limited on the cost-effectiveness of aspirin or LMWH chemoprophylaxis after knee arthroscopy. Therefore, this study aimed to examine the cost-effectiveness of using aspirin and LMWH to prevent VTE after knee arthroscopy. Given the high reported incidence of symptomatic VTE after knee arthroscopy, we hypothesized that both aspirin and LMWH are cost-effective in preventing symptomatic VTE and direct financial costs associated with treating VTE using a “break-even” analysis.

## Methods

A break-even analysis—initially described by Hatch et al^10^ (equation 1)—has been used as an economic model for determining the cost-effectiveness of prophylactic interventions to prevent more costly adverse outcomes. This equation produces the final break-even adverse outcome rate necessary to make a protocol cost-effective based on the initial adverse outcome rate, the total cost of treatment for that adverse outcome, and the cost of a specific prophylactic treatment or protocol. Calculating the difference between the initial and final break-even adverse outcome rate yields the absolute risk reduction (ARR), which is the percentage by which a drug must reduce the initial adverse outcome rate to economically justify its use. With the ARR, the number needed to treat (NNT) can also be calculated to provide a threshold of cases that could be performed using a given prophylactic agent to break even on cost when 1 symptomatic adverse outcome is prevented. In this study, symptomatic VTE was selected as the adverse outcome. Aspirin and LMWH were assessed separately as prophylactic agents to prevent symptomatic VTE.

To obtain values for our modified equation, we calculated the cost of treating a symptomatic VTE from purchasing records of our institution. The Medline database was then queried for research articles published within the past decade that reported VTE rates after knee arthroscopy. The lowest and highest rates found in our search were then used for analysis.

To objectify the incidence of VTE, the TriNetX research database was queried using the International Classification of Diseases codes I26 (pulmonary embolism), I82.62 (acute embolism and thrombosis of deep veins of upper extremity), and I82.64 (acute embolism and thrombosis of deep veins of lower extremity), and using the Current Procedural Terminology codes 29888 (arthroscopically aided anterior cruciate ligament repair/augmentation or reconstruction), 29882 (arthroscopy, knee surgical; with meniscal repair medial or lateral), 29875 (arthroscopy knee, surgical; synovectomy, limited), 29876 (arthroscopy, knee, surgical; synovectomy, major, 2 or more compartments), and 29877 (arthroscopy knee, surgical; debridement/shaving of articular cartilage). Only patients who did not receive any form of anticoagulation or aspirin therapy for up to a month after surgery were included in the final analyses. Patient characteristics were also collected (Table 1). We used an open-access retail pharmaceutical database to obtain the average retail price for 3 weeks of treatment with aspirin 325 mg twice daily (BID), enoxaparin 30 mg BID, and enoxaparin 40 mg once daily (QD).^6^ We chose these formulations as they are routinely used at our institution.

(Equation 1) $$S_{\mathrm{total}}\;\times \;C_{t}\;\times \;VR_{i}=\left(S_{\mathrm{total}}\;\times \;C_{d}\right)+\left(S_{\mathrm{total}}\times \;C_{t}\;\times \;VR_{f}\right)$$

Solving for *VR_f_* yields:

$$VR_{f}=\frac{\left(VR_{i}\times C_{t}\right)-C_{d}}{C_{t}}$$

This equation was used to calculate the break-even VTE rate,

where S_total_ = total annual surgeries; C_t_ = total cost of treating a VTE; C_d_ = cost of drug(s); VR_i_ = initial VTE rate; and VR_f_ = break-even VTE rate.

(Adapted from Hatch MD, Daniels SD, Glerum KM, Higgins LD. The cost-effectiveness of vancomycin for preventing infections after shoulder arthroplasty: a break-even analysis. *J Shoulder Elbow Surg*. 2017;26(3):472-477.^10^)

**Table 1:** Patient Characteristics^*a*^

Patient Characteristics	
Mean age, years	33.7 ± 16.1
Sex	
Female	47,233 (48)
Male	50,160 (51)
Unknown	1337 (1)
Ethnicity	
Not Hispanic or Latino	70,809 (72)
Hispanic or Latino	8578 (9)
Unknown Ethnicity	19,343 (19)
Race	
White	65,560 (66)
Black or African American	12,278 (12)
Asian	1993 (2)
American Indian or Alaskan Native	490 (1)
Native Hawaiian or Other Pacific Islander	147 (<1)
Unknown	18,262 (19)

a Data are presented as mean ± SD or n (%).

## Results

At our institution, the estimated cost of treating a symptomatic VTE was found to be $9407. The product cost for a 3-week course of aspirin (325 mg BID), enoxaparin (30 mg BID), and enoxaparin (40 mg QD) were found to be $1.86, $188.72, and $99.99, respectively (Table 2). A VTE rate of 0.34% previously described by Krych et al^14^ was used as the low rate, while the high rate of 10.9% was adopted from Reynolds et al.^21^ Using the TriNetX research database, a total of 98,730 patients who underwent arthroscopic knee surgery from 2011 to 2021 without postoperative anticoagulation or aspirin therapy were identified. Of these patients, 849 (0.86%) developed a VTE. Aspirin (325 mg BID) was found to be cost-effective at the low, TriNetX, and high VTE rates if the initial rate decreased by an ARR of 0.02% (NNT = 5058). Enoxaparin (30 mg BID) and enoxaparin (40 mg QD) were cost-effective at the high initial VTE rate if their initial rates decreased by ARRs 2.01% (NNT = 50) and 1.06% (NNT = 94), respectively. However, at the low and TriNetX initial VTE rates, the 2 doses of enoxaparin were not cost-effective, as their final break-even rate exceeded the initial VTE rate (Table 3).

**Table 2:** The Mean Cost of VTE Prophylactic Agents Given for 3 Weeks^*a*^

Drug	Dose, mg	Frequency	Route of Administration	Mean Price, $ (US Dollars)
Aspirin	325	BID	PO	1.86
Enoxaparin	30	BID	SQ	188.72
Enoxaparin	40	QD	SQ	99.99

a All prices were obtained from an online drug database.^6^ BID, twice daily; PO, by mouth; QD, once daily; SQ, subcutaneous; VTE, venous thromboembolism.

**Table 3:** Cost-Effectiveness of Chemoprophylactic Agents in Knee Arthroscopy^*a*^

Drug, mg	Initial VTE Rate, %	Final VTE Rate, %	ARR, %	NNT
Aspirin 325	^*b*^0.34	0.32	0.02	5058
	^*c*^0.86	0.84	0.02	5058
	^*d*^10.90	10.88	0.02	5058
Enoxaparin 30	0.34	−1.67	2.01	50
	0.86	−1.15	2.01	50
	10.90	8.89	2.01	50
Enoxaparin 40	0.34	−0.72	1.06	94
	0.86	−0.20	1.06	94
	10.90	9.84	1.06	94

a All prices were obtained from an online drug database (Table 1). ARR, absolute risk reduction; NNT, number of patients who need to be treated; VTE, venous thromboembolism.

Our institution assumes that the cost of treating a VTE is $9407 (US Dollars).

b Low VTE rate obtained from the literature.^14^

c TriNetX VTE rate.

d High VTE obtained from the literature.^21^

## Discussion

VTE chemoprophylaxis after knee arthroscopy remains debated.^4^ This controversy stems from the variability in reported VTE incidence after knee arthroscopy, with studies^14,17,21^ citing rates as low as 0.34. As a result, not only has the utility of VTE chemoprophylaxis been questioned but so has its cost-effectiveness.^21^ Our study is the first to examine the cost-effectiveness of routine use of commonly used chemoprophylactic medications to prevent VTE after knee arthroscopy. At our determined rates of VTE for common knee arthroscopy procedures and cost of VTE treatment, aspirin 325 mg BID (at a cost of $1.86 for a 3-week course) is a cost-effective method of VTE prophylaxis, while enoxaparin 30 mg BID (at a cost of $188.72) and enoxaparin 40 mg QD (at a cost of $99.99 for a 3-week course) are only cost-effective (at a cost of $96.08 for a 3-week course) at our highest initial VTE rate.

The break-even cost-effectiveness model allowed us to calculate the NNT, which is useful in framing the practicality of each chemoprophylactic agent. For example, we calculated that aspirin has an NNT of over 5000 patients. This means that just 1 VTE would need to be prevented after 5000 knee arthroscopies with the use of aspirin prophylaxis for aspirin to break even on cost. While the NNT is useful, we must caution against interpreting it in isolation. An example from our study is the NNT of 50 patients for enoxaparin 30 mg BID. Preventing 1 VTE every 50 arthroscopies appears advantageous; however, the majority of contemporary literature reports that VTE occurs less frequently than every 50 arthroscopies. Therefore, enoxaparin 30 mg BID, at least in economic terms, cannot break even except at very high rates of VTE or very low costs of enoxaparin. The NNT supplements our findings but must be considered within the context of the ARR.

While anticoagulant agents have historically been the drug of choice in orthopaedic surgery, recent studies have investigated the efficacy of aspirin for VTE prophylaxis. In particular, aspirin has been found to be an effective method of VTE prophylaxis in obese patients undergoing revision total hip or knee arthroplasty.^22^ Furthermore, in 2021, Hasan et al^9^ performed a 1-year observational study among adults undergoing elective knee arthroplasty and found aspirin to be as effective as apixaban for preventing VTE and hospital readmission. Likewise, a study by Hood et al^11^ found that aspirin was noninferior to other forms of anticoagulation at preventing postoperative VTE or death in patients undergoing total knee arthroplasty.

There is limited information on the role of aspirin in arthroscopy to support or refute its use. In 2015, Kaye et al^13^ performed a single-center randomized controlled trial in a relatively small number of low-risk patients (N = 170) undergoing knee arthroscopy and reported no deep vein thrombosis detected by ultrasonography in either arm of their study after 2 weeks of treatment with aspirin 325 mg BID. A 2022 study by Reynolds et al^21^ reported reduced VTE incidence in patients undergoing knee arthroscopy when given aspirin (325 mg BID) or LMWH (40 mg QD) compared with historical rates of VTE. Interestingly, in 2021, Qin et al^20^ reported that arthrofibrosis after anterior cruciate ligament repair occurred in 1.9% of patients who received aspirin, compared with 4.3% of patients who received chemoprophylaxis with agents other than aspirin, albeit their study did not comment on the effectiveness at preventing VTE.

There are a few important considerations that can be derived from this study. First, the major determinant of cost-effectiveness was the cost of the drug. At its average price, a 3-week course of aspirin 325 mg BID was much cheaper than enoxaparin 30 mg BID and enoxaparin 40 mg QD, allowing aspirin to achieve cost-effectiveness at all initial VTE rates. While we selected aspirin 325 mg BID for our analysis, recent literature has reported aspirin 81 mg BID to be equally as effective as aspirin 325 BID for VTE prophylaxis in joint arthroplasty.^23^

As such, aspirin 81 mg BID will be just as cost-effective and may be even more cost-effective than aspirin 325 mg BID because of its lower product cost. Second, when the product cost of enoxaparin (30 mg BID) and enoxaparin (40 mg QD) is lowered, they can both achieve cost-effectiveness (Table 4). Finally, these break-even points may be lowered—we utilized $9407 as our cost for treating a VTE, which accounts for the costs associated with immediate inpatient hospitalization and treatment. However, this value may be significantly higher given that the average acute VTE-related medical costs have been reported as high as $15,000.^7^ Lin et al^16^ reported that this price increases to $24,282 in 1 month and $38,591 in 1 year for patients with recurrent VTE.

**Table 4:** Enoxaparin Can Become Cost-Effective at Lower Prices^*a*^

Drug, mg	Initial VTE Rate, %	Cost of Treating VTE, $	Cost of Prophylactic Regimen, $	Final (Break-Even) VTE Rate, %	ARR, %	NNT
Enoxaparin 30	0.34	9407	188.72	−1.666	2.01	50
	0.34	9407	163.72	−1.400	1.74	57
	0.34	9407	153.72	−1.294	1.63	61
	0.34	9407	143.72	−1.188	1.53	65
	0.34	9407	133.72	−1.081	1.42	70
	0.34	9407	123.72	−0.975	1.32	76
	0.34	9407	113.72	−0.869	1.21	83
	0.34	9407	103.72	−0.763	1.10	91
	0.34	9407	93.72	−0.656	1	100
	0.34	9407	83.72	−0.550	0.89	112
	0.34	9407	73.72	−0.444	0.78	128
	0.34	9407	63.72	−0.337	0.68	148
Enoxaparin 40	0.34	9407	99.99	−0.723	1.06	94
	0.34	9407	89.99	−0.617	0.96	105
	0.34	9407	79.99	−0.510	0.85	118
	0.34	9407	69.99	−0.404	0.74	134
	0.34	9407	63.72	−0.337	0.68	148
	0.34	9407	13.72	0.194	0.15	686

a ARR, absolute risk reduction; NNT, number of patients need to be treated; VTE, venous thromboembolism.

While our study provides a useful framework for any physician to evaluate cost-effectiveness in one’s practice, there are several limitations. First, the cost of treating symptomatic VTE and the cost of VTE chemoprophylaxis certainly vary by institution and region. Second, while our study did provide an objective value for VTE rates, our data were collected from a large database that relies on administrative coding, which may fail to adequately detect the true rate of VTE. Additionally, we only considered symptomatic VTEs, when in reality, asymptomatic VTEs that develop may also prove to have serious clinical and economic implications, albeit the ramifications of asymptomatic VTEs have yet to be determined.

Third, even though we reported varying rates of VTE in multiple types of arthroscopic procedures, our modeling only accounts for averages and does not capture patient-specific factors, such as patient characteristics and comorbidities. Fourth, our analysis likely underestimates the true fiscal effect of treating a symptomatic VTE, as we only considered hospital-related expenses in our analysis. However, added nonhospital expenses incurred by the patient would only further increase the total cost of treating a symptomatic VTE, increasing the likelihood that a VTE prophylactic agent would be cost-effective. Finally, our study fails to capture the risk and expenses associated with bleeding complications, while also not considering the cost of gastrointestinal (GI) protective medications that some surgeons may prescribe. However, research has shown that bleeding risk is minimal, and a recent study by Grosso et al^8^ reported that aspirin is not only safe in patients with a history of GI issues undergoing total joint arthroplasty but also found that there was no associated increased risk of postoperative GI bleeds.^1,19,24^ Nevertheless, if a patient goes on to develop a postoperative bleed, previous literature has estimated the cost of treating an acute GI bleed to be $600,026; however, this added expense would not affect the ability of a VTE prophylactic agent to remain cost-effective.^25^

Despite these limitations, we believe that our model can provide a conceptual framework that physicians can easily apply to their practice to further improve patient care. Furthermore, our model provides data on cost-effectiveness that would be unattainable in a clinical study. For example, to determine an ARR of 0.01%, with 80% power and *P* < .05, the sample size would need to include 61,465,600 patients.

## Conclusion

Based on our results, we believe that a 3-week course of aspirin 325 mg BID at its average retail price is a cost-effective option to reduce VTE in low-risk patients undergoing arthroscopic knee surgeries. Furthermore, we believe that if enoxaparin (30 mg BID or 40 mg QD) can be obtained for a lower cost, or if the patient has an elevated risk of VTE, its use can be economically justified. Even though there is no clear consensus on chemoprophylaxis after arthroscopy, we believe that the potential economic burden that a VTE produces should be considered and prompt further investigation. The decision to use enoxaparin or aspirin in the postoperative period should be shared between the physician and the patient, and the patient’s underlying risk factors should be taken into consideration.
